# Stigma and discrimination related to mental health and substance use issues in primary health care in Toronto, Canada: a qualitative study

**DOI:** 10.1080/17482631.2020.1744926

**Published:** 2020-03-31

**Authors:** Maureen A. Murney, Jaime C. Sapag, Sireesha J. Bobbili, Akwatu Khenti

**Affiliations:** a Interdisciplinary Centre for Health & Society, University of Toronto Scarborough, Toronto, Canada; b Department of Community Health Sciences, Centre for Global Public Health, Max Rady College of Medicine, University of Manitoba, Canada; c WHO / PAHO Collaborating Centre for Addiction and Mental Health, Institute for Mental Health Policy Research, Centre for Addiction and Mental Health (CAMH), Canada; d Clinical Public Health Division, Dalla Lana School of Public Health, University of Toronto, Toronto, Canada; e Department of Public Health and the Department of Family Medicine, Pontificia Universidad Católica De Chile, Santiago, Chile; f Social & Behavioural Health Sciences Division, Dalla Lana School of Public Health, University of Toronto, Toronto, Canada

**Keywords:** Mental illness, addiction, stigma, recovery, Canada, primary health care

## Abstract

Purpose: Community Health Centres (CHCs) are an essential component of primary health care (PHC) in Canada. This article examines health providers’ understandings and experiences regarding stigma towards mental health and substance use (MHSU) issues, as well as their ideas for an effective intervention to address stigma and discrimination, in three CHCs in Toronto, Ontario. Methods: Using a phenomenological approach, we conducted twenty-three interviews with senior staff members and peer workers, and three focus groups with front-line health providers. Ahybrid approach to thematic analysis was employed, entailing a combination of emergent and *a priori* coding. Results: The findings indicate that PHC settings are sites where multiple forms of stigma create health service barriers. Stigma and discrimination associated with MHSU also cohere around intersecting experiences of gender, race, class, age and other issues including the degree and visibility of distress. Clients may find social norms to be alienating, including behavioural expectations in Canadian PHC settings. Conclusions: Given the turmoil in clients’ lives, systematic efforts to mitigate stigma were inhibited by myriad proximate factors that demanded urgent response. Health providers were enthusiastic about implementing anti-stigma/recovery-based approaches that could be integrated into current CHC services. Their recommendations for interventions centred around communication and education, such as training, CHC-wide meetings, and anti-stigma campaigns in surrounding communities.

## Introduction

There is increasing recognition worldwide that mental health is a vital aspect of overall health (Arboleda-Flórez & Saraceno, 2001; Kirmayer & Pedersen, 2014; Patel & Chatterji, 2015). Indeed, mental health and substance use (MHSU) issues represent a substantial contribution to the global burden of disease (Vigo et al., 2016). In Ontario, Canada, the number of patients with mental health issues, as well as the cost of treating them, are increasing (Rehm et al., 2006; Silveira et al., 2016; Sunderji et al., 2018). Primary health care (PHC) settings are well-situated for facilitating the early detection of MHSU issues, and for providing affordable treatments and follow-up care, because general practitioners are responsible for a significant proportion of mental health care (Borges et al., 2016; Ivbijaro, 2012). In Ontario, most patients obtain mental health care solely from a general practitioner (Arboleda-Flórez & Saraceno, 2001; Statistics Canada, 2013). Furthermore, between 25 and 30% of patients in PHC settings can be expected to suffer from mental health-related issues, though less than half of these cases are detected (Stuart et al., 2012). However, PHC settings may become sites where clients must contend with various forms of stigma and discrimination, including forms perpetuated by health providers.

Stigma and discrimination exist worldwide, relating to both mental health (Mascayano et al., 2016; Pescosolido et al., 2008; Stuart et al., 2012) and substance use (Corrigan et al., 2017; Room, 2005; Van Boekel et al., 2013). Many factors influence the under-detection of mental illness in PHC settings, including differing socio-cultural expressions of mental illness (Brijnath & Antoniades, 2018; Kirmayer et al., 2017; Kirmayer & Pedersen, 2014), and the stigma associated with mental illness (Michels et al., 2006; Sapag et al., 2018). Stigmatizing attitudes and practices among health providers are well-documented (Corrigan, 2004; Schulze, 2007), and clients of mental health services have reported stigmatizing treatment from both general practitioners and psychiatrists (Thornicroft et al., 2007). According to Stuart et al. (2012), stigma and discrimination can be experienced in health care settings by, for example, being threatened with coercive treatment, being provided with insufficient information, being regarded as lacking the capacity for responsible action, and being patronized or humiliated.

Evidence suggests that the integration of mental health services into PHC provides a number of advantages including: 1) reduced stigma; 2) improved prevention and detection of mental health problems; 3) reduced chronicity and improved social integration; 4) human rights protection; 5) better health outcomes; and 6) improved human resources capacity for mental health care (Sunderji et al., 2018; WHO & WONCA, 2008). Various international and national programmes have been developed to ameliorate stigma relating to mental illness, and the number of programmes is growing. However, there remains a dearth of knowledge about stigma and discrimination relating to MHSU issues *within* PHC sites (Shim & Rust, 2013). Furthermore, few studies have examined the development and implementation of processes or interventions to ameliorate MHSU-related stigma and discrimination among health providers within PHC settings (Khenti et al., 2017a; Mehta et al., 2015; Sapag et al., 2018). Stuart et al. (2012) maintain that when developing anti-stigma programmes, it is important to target local needs and the specific behaviours of well-defined groups, such as health professionals, youth, police, and policy makers. Moreover, they claim that “health professionals present one of the most strategically located, yet challenging, groups for anti-stigma efforts” (Stuart et al., 2012, p. 158).

Within Ontario, an important component of PHC is offered by Community Health Centres (CHCs), where health professionals with formal degrees, such as nurses, nurse practitioners, physicians, social workers and dieticians, collaborate with other personnel such as community outreach workers and peer workers, to provide interdisciplinary care in one location. For the purposes of this paper, we refer to all the above groups under the umbrella term of “health providers,” as they all provide valuable care within CHC settings. The uniqueness of CHCs lies in the fact that they are the only PHC setting in Ontario that addresses the social determinants of health (SDOH) by integrating health promoters, harm reduction staff, community outreach and peer workers, and others into their model of care (Association of Ontario Health Centres, 2009). These health providers focus on community development but also respond to health problems caused by social, environmental, and economic factors and develop customized services and programmes for the diverse communities they serve. Because CHCs are specifically designed for eliminating systemic barriers to access, such as poverty, racism, heterosexism, language discrimination, ableism and other forms of social exclusion, they are more effective for those who have traditionally faced difficulties in accessing and benefiting from traditional health care (Association of Ontario Health Centres, 2009).

CHCs provide services to many patients with mental illness and chronic health conditions (Glazier et al., 2012). Services for MHSU are based on the concept of recovery. Since the 1990s, recovery has been viewed as a crucial concept and process for helping people with MHSU issues to heal; however, that healing might be conceptualized differently under differing circumstances (Anthony, 1993; Poole, 2011). Over time and with much debate between clients/consumers, health providers, and policy makers, recovery has come to mean “a complex, dynamic, and enduring process rather than a biological end-state described by an absence of symptoms” (White et al., 2005, p. 234). Recovery-oriented practices—including the promotion of interpersonal skills, working collaboratively, and sharing knowledge—are regarded as essential for contending with MHSU issues (Cleary & Dowling, 2009; Gronholm et al., 2017). Furthermore, there is a recognized need for effective anti-stigma interventions with a strong focus on recovery (Gilburt et al., 2013).

Capacity-building work conducted by the Centre for Addiction and Mental Health (CAMH) with CHCs in Ontario has demonstrated a need to address the stigma and discrimination associated with MHSU problems within PHC settings (Khenti et al., 2016, 2017b). Indeed, there has been an expressed interest among CHC personnel to address the challenges of stigma and discrimination. As a result, an applied, action-research project was developed in close collaboration with CHC partners; this paper presents the results of a qualitative study nested within the larger, mixed-method project. The aim of this qualitative study was, firstly, to explore the stigma and discrimination faced by people with MHSU issues within PHC settings in Toronto, Ontario, Canada. Secondly, the intent of the study was to inform the design of an effective and portable intervention to address stigma and discrimination. Three CHCs in Toronto contributed to the project; these organizations varied in terms of geographical location within the city, client groups served (e.g., youth, sex workers, different ethnic groups), and programmes offered.

### Conceptualizing stigma

Stigma was initially theorized by Goffman (1963) as an individual’s experience of disgrace, a kind of mental identification, a “spoiled identity” resulting from processes of discrimination. Over recent decades, researchers have recognized structural discrimination and socio-economic repercussions for stigmatized people. Broadly speaking, stigma affects levels of stress, life chances, and can add to the burden of disease or disability (Hatzenbuehler et al., 2013; Link & Phelan, 2006). While social, economic, and political influences “[shape] the distribution of stigma within a social milieu” (Kleinman & Hall-Clifford, 2009, p. 418), it is important to keep in mind that factors affecting stigma and discrimination are not simply additive in experience or effect (Crenshaw, 1989). For example, racism and sexism interact or intersect for racialized women, resulting in particular experiences of subordination for women of colour, compared with white women or men of colour. Accordingly, in this paper we conceptualize “intersectional stigma” (Logie et al., 2011) as operating through a complex, relational terrain of identity, experience, and aspects of privilege (e.g., race; gender; housing status; visibility of a practice such as substance use, or visibility of a disease or disability; and so on) that not only influence peoples’ immediate health and well-being, or their future life chances, but also their ongoing ability to navigate a multitude of settings.

## Methods

### Design

In research on stigma and discrimination, qualitative methods can deepen our understandings of the lived experiences of stigma, and the processes of power through which stigma is created, reproduced, altered, and reduced (Link et al., 2004, cf. Yang et al., 2007). As such, qualitative methods provide key information for anti-stigma programme developers (Hamilton et al., 2016; Stuart et al., 2012). A phenomenological approach, which considered both descriptive and interpretive aspects of human experience (Reiners, 2012), was employed for the qualitative component of this project. In other words, investigators sought not only to uncover the experiences that health providers encountered in relation to MHSU issues with clients, but also sought to illuminate the meanings that health providers assigned to those experiences.

### Participants

Participants were chosen via purposive sampling (Patton, 2002). Semi-structured interviews were carried out with 23 staff members across three CHCs (14 senior staff and 9 peer workers). Senior staff members had in-depth knowledge of the organization, as a whole, and represented a range of roles within the CHCs. This group of participants included representatives from: i) management, such as the executive director or equivalent; ii) clinical teams; iii) community health programmes; and others based on context (e.g., finance). Peer workers fulfil various roles at the CHCs and also have experiential knowledge of MHSU issues. Interviews with peer workers represented as much diversity as this small number of participants would allow for, in terms of age, ethnicity, gender, roles within the CHCs, location of work (in the Centres versus out in the communities), and how long they had been involved with the organization (as staff and/or as clients). In addition, three focus groups were carried out with health providers who worked directly with clients; 10 to 12 people attended each group. Focus group participants represented a number of staff roles having direct contact with clients, including front-line reception, social workers, physicians, nurses and others.

### Data collection

Qualitative data were gathered through interviews and focus groups, and focused on stigma and discrimination towards people with MHSU issues, among CHC health providers (including peer workers) and especially within CHC-related contexts. In developing the interview and focus group guides, our approach was to explore the client populations that health providers served, the services they offered, how these services connected with the daily needs of clients, while eliciting examples, and then focusing on the intervention. The interview and focus group questions were guided by two overarching research questions: i) What are providers’ understandings of the problem? and ii) What are providers’ ideas for an intervention? More specific research questions included: “What are the main mental health or substance use problems for your client populations?” and “Are there factors other than MHSU problems, operating at the community level, that might affect experiences of stigma?” Logistical meetings were held with CHC liaison representatives to determine who should be invited to participate, and locations and schedules for carrying out interviews and focus groups. All interviews and focus groups were audio-recorded, except for one interview where the participant was uncomfortable with the recording technology and preferred only note-taking. Interviews and focus groups were conducted by the first author, while detailed notes were taken for all meetings by a research assistant who then transcribed the recordings.

The project was originally planned so that a small number of staff would be interviewed and one focus group would be conducted at each CHC. After completing this initial research phase, a key stage in the process took place in the form of a symposium held with CHC project liaisons, CHC staff including many of the research participants, and an international advisory panel of experts on stigma. The symposium offered the opportunity to review our data anlysis and interpretations, a process known as “member checking” (Connelly, 2016; Lincoln & Guba, 1985; Patton, 2002), as well as engage in knowledge translation. Based on the symposium recommendations, we determined that additional critical data could be obtained by conducting interviews with “peer workers.” Given their personal experiences with MHSU problems and their knowledge of health care settings, it was expected that peer workers would provide deep knowledge of stigmatizing attitudes and experiences encountered in and around the CHCs, as well as health care contexts more generally. Organizational meetings were held with CHC liaisons and/or other CHC representatives, who then invited peer workers for interviews. Thus, interviews with peer workers offered opportunities to confirm, challenge, and deepen researchers’ understandings.

### Data analysis

A hybrid approach to thematic analysis was used for coding interview and focus group data (Rubin & Rubin, 2005, pp. 201–23). This entails a combination of: a) open coding, wherein coding is done as the analyst reads along to capture the ideas that emerge, and b) coding for a topical study, wherein the analyst develops codes for concepts and themes derived from the interview questions and the literature. For example, codes that emerged from the data included concepts such as [challenging behaviours] and [mainstream health care], while *a priori* codes derived from the interview questions and literature included [gender] and [training]. Emergent and *a priori* codes were reconciled, combined, and integrated into a single coding scheme with refined definitions developed for consistency. After coded data were sorted, grouped, and further analysed, they were linked to concepts relating to stigma and intersectional theory (e.g., Crenshaw, 1989; Link & Phelan, 2006), in order to illuminate the ways that participants’ ideas and experiences were enmeshed in broader social and structural frames. In particular, the researcher searched for ideas or experiences that invoked frustration and challenge for participants. Our conceptual framework around intersectional stigma emerged during this analytic stage; this notion served as the lens through which we were able to further interpret the findings.

### Ethical considerations

This study was reviewed and approved by the Research Ethics Board at the Centre for Addiction and Mental Health. Informed consent was obtained from all participants; letters of consent were read by all participants and subsequently signed.

## Findings

Participant narratives revealed multiple, interconnecting forms of stigma and discrimination encountered in CHCs by a diverse clientele contending with issues relating to MHSU. Although participants, as health providers, attempted to manage these various challenges, they did not always feel well-equipped with the necessary skills and resources to fully respond. In this section, we first provide a broader overview of the contextual factors that shaped forms of stigma and discrimination encountered by clients. We then focus on prominent themes that emerged from the narratives that tie into these contextual factors, including socio-cultural beliefs, differences in stigma and discrimination experienced with mental health versus substance use, alienation in PHC settings, and issues around communication. Finally, we shift our thematic focus to the various ways that participants responded to MHSU-related stigma and discrimination, and their ideas for interventions in PHC settings to decrease this stigma and discrimination.

### Contextual factors and social determinants of health

Overall, research participants framed ideas of stigma and discrimination in relation to a host of contextual factors that CHC clients encountered. The clients they referred to included Indigenous people, immigrants and refugees, street-involved youth, and transgender people. Health care providers confronted a broad constellation of issues in their delivery of health services. Their clients experienced disorders relating to mental health, substance use, and other health problems. Clients suffered from depression, anxiety disorders, bipolar disorder, schizophrenia, post-traumatic stress disorder, and attention deficit disorders. Furthermore, their clients used substances such as crack cocaine, opiates (mostly heroin, methadone, morphine, Dilaudid, and Oxycontin), methamphetamine, ketamine, cannabis, and alcohol. These problems, participants explained, were complicated by HIV, Hepatitis C, diabetes, and other diseases.

Participants also emphasized the significance of social determinants of health in relation to clients’ experiences with stigma. These social determinants included poverty, housing status (whether homelessness or insecure housing), lack of food security and poor nutrition, gender bias, age bias (e.g., against homeless youth), racism, ethnocentrism, language barriers, access to training and jobs, and types of work (e.g., sex work). Particular emphasis was placed on the relationship between MHSU problems and trauma (including intergenerational trauma); violent or otherwise abusive family history; violence in present-day interactions; fear and anxiety (e.g., over owing money to dealers and fearing repercussions, or worrying that one’s children would be taken away); struggles with a poor sense of self or low self-esteem; isolation; and dislocation, especially with respect to familial experiences with residential schools, or other forms of “Crown involvement” (i.e., governmental intervention).

For example, at two of the three CHCs, participants described Indigenous clients who were under-housed or homeless, and who had endured cultural trauma and personal traumatic experiences. Many of these clients also experienced long-term poly-substance use and mental health issues. Immigrant and refugee populations at all three CHCs were reported to experience multiple obstacles, including language barriers, and fears that their citizenship status might be revealed. Street-involved youth were characterized as experiencing particular difficulties as well. In addition to struggles with familial trauma, poverty, violence, troubles with the police, and other problems commonly encountered on the street, youth were depicted as dealing with a lack of adult care and guidance while growing up and developing their identities.

Referring to harm reduction clients, participants discussed how many of the women and some of the men dealt with a history of sexual abuse, physical abuse, and neglect, as well as racism or other forms of discrimination. Sex workers, participants maintained, confronted problems relating to sexual assault, rape, physical assault, or being robbed.

Transgender people were described as enduring virulent forms of stigma and discrimination. Especially for this group, participants noted that stigma and discrimination in the neighbourhood around the CHC affected accessibility. That is, potential clients would not walk through a gauntlet of harassment in order to obtain health services.

### Socio-cultural beliefs and stigma regarding mental health or substance use

Some participants described stigma in terms of cultural beliefs. For example, they explained that problems can arise when the client’s knowledge or experiences did not coincide with North American medical understandings. As one CHC clinician stated, there can be “a lot of other cultural pieces that go with [the health issue] in terms of their belief as to what’s impacting them.”

In discussing family relationships and stigma relating to mental illness, a senior staff member claimed:

A lot of people have a lot of stigma around family. There is a perception that mental illness is hereditary, so if one person in the family has it then it can, if you will, pollute the rest of the family unit, so that’s something you’re going to keep … hidden away, and not going to share with your neighbours.

Participants cautioned that providers may also make incorrect assumptions about a client’s culture or place of origin. One example given was the assumption that West African men are more likely to drink alcohol than use drugs, as if drugs are uncommon in Africa. As a result, in one community, there was a lack of services for West African men who used crack cocaine.

Participants reported difficulties being attentive to culture and class, and expressed concern about imposing a Canadian, white, middle class morality in their recommendations to clients about how to improve their life circumstances (with respect to education, having a career trajectory, buying a home, and so on). A clinician explained, “We’re so afraid now of suggesting [these outcomes to a client] … I wouldn’t want to imply that this person should do that, because now I’m putting white, middle class values on this person.”

On the other hand, clinicians commented upon providers’ inclinations to presume Caucasians universally share the same cultural, social, and economic advantages (e.g., a Polish versus an Irish immigrant). One clinician observed a conflation of the middle class with white-ness:

If you look at middle class people, they’re broad, diverse people with different cultures, different countries … different ethnicities, who are all able to work and do well and have productive lives, and there’s lot of white people who are poor, and live in bad housing and on the streets. … We sort of pretend that white people are all happy, healthy people living productive lives.

Participants also commented upon the ways that their own cultural understandings can come into play more strongly, and potentially cause discrimination, depending upon training and experience. One senior staff member remarked:

… the reality is, if [MHSU issues are] not your core work, then [your] cultural background will take up more of your understanding. And then there are people within [the organization] who deeply accept the cultural norm, and others who reject it. We’re no different than any other microcosm. We have a range of [cultural beliefs about mental health and substance use].

Thus, providers grapple with multiple ambiguities as they attempt to balance their own cultural beliefs and assumptions, the experiences of their marginalized clients, and medical knowledge and practice.

### Mental health versus substance use

While there may be similarities in the stigmas of mental health and substance use, since sufferers in both instances “tend to get blamed for their behaviours,” research participants often differentiated the two. Their distinction was based in part on the idea of intention; in the case of substance use, the user is popularly believed to be *choosing* an unhealthy, dangerous “lifestyle” as part of an irresponsible but nonetheless conscious decision-making process, while a person with mental illness is regarded as a victim of circumstance. Indeed, participants noted that enmity is often directed at substance users, even by those who claim to support the disease model of addiction. Differences in attitudes, then, can be summarized as pity versus hostility and judgement. The situation for the sufferer is then compounded if there are both mental health and substance use issues.

According to health providers, experiencing stigma and feelings of low self-esteem are deeply entangled with the illegality of drug use. A senior staff member explained:

With substance use and crack cocaine in particular which we do see a lot of, there’s a lot of negative beliefs about substance users. In the community, people are assumed to be criminals, degenerates, responsible for social ills in the neighbourhood …. There’s a stigma just associated with drug users, there’s another layer to it with it being criminalized.

Similarly, a peer worker remarked:

If you need to do your drugs, you need to do it … it can’t just be wished away. You’re torn between [wishing to stop and feelings of low self-esteem], then you still have to come out and acquire it … make sure it’s real, and find a place to use it without getting arrested by the police, and without the neighbour seeing you … It’s chaotic, absolute chaos.

Anger and frustration can also lead substance-using clients to consume more drugs in order to cope. Such experiences can lead to difficulties at the CHCs; clients may arrive angry and frustrated, and can become volatile or aggressive, thus requiring a crisis worker or other health provider to de-escalate the situation.

A key factor with substance use-related stigma is gender; for women, stigma relating to substance use is often perceived and experienced in relation to their reproductive roles. As a peer worker remarked, “If you see a man drunk on the street, it’s one thing. If you see a woman drunk on the street, it’s another. It’s just the way society is.” This perspective is common around the globe (Murney, 2009; Waterson, 2000; Wilsnack et al., 2005). A senior staff member commented:

The risks of losing custody for your child are huge for women. The risks for disclosing your substance use patterns are far more punitive for women than men. So they have a whole lot more to risk by stepping out and disclosing their history around substance use. The second thing is that substance use, in particular around crack cocaine, tends to also be associated with other risks including sexual risk, including … increased activity and participation in sex work, which is another highly stigmatized piece … so I think it’s a whole lot more challenging for women to step out and disclose …

However, participants reported MHSU-related stigma among men relates to their gender roles as income earners. Another senior staff member remarked:

Mental illness is considered to be a really big vulnerability. I think this is where there’s that additional risk to men, so the drug trade is associated with an economic … “okay, I’m a hustler, I’m out conducting business, this is purely business, I have power and I’m in control of what happens.” Within the population of men who are active in the drug trade, the stigmas around substance use are increased ten-fold because it is also a financial risk [for] you and your financial network …

In summary then, these various contextual factors can lead to multiple, overlapping experiences of stigma that may compound the MHSU issues themselves, and in turn affect interactions within CHC settings.

### Alienation in health care settings

Participants were acutely aware that immigrant and refugee clients may encounter CHC services as alienating, and this was reflected in how they tried to communicate and respond in their health delivery practices. For example, participants discussed the complexity of trying to involve a client in decision-making processes regarding their health, when this countered the client’s experiences of medical practice in their home countries, where physicians exercised authority by dictating a course of action.

Like if you look at some countries, if you come to a doctor … a doctor is supposed to know everything. If you look at our interaction and what we’re trying to do, you want to involve the client or the patient to be part of the process. You know, that’s not the culture, that’s not their understanding, to be a part of [the process] … “Tell me what’s wrong. And fix it.”

In addition, participants outlined the alienating effects arising from norms established in CHCs, as in other Canadian health care institutional settings. For instance, clients were expected to wait unobtrusively in a room with other clients without shouting or making expansive gestures, and it was expected that they would not publicly engage in acts that are commonly deemed to be private, such as open-wound care. Participants were certainly aware of how such norms conflicted with clients’ needs, but sometimes struggled with how to reconcile this conflict. As one intake worker recounted:

There was an instance where our client felt that I thought he was dirty because he wanted to change his leg wounds in our front lobby. … I’d worked so hard to organize home care for him. … He had someone at home that he was actually standing up while he was sitting in our lobby, and I was just so frustrated that you do so much for this guy and he doesn’t engage in it. The other thing is legally his wound is beyond my scope of practice. I’m not supposed to be treating HIV-infected wounds; it’s just not what I’m allowed to do. But he wanted to change it himself. He said, “Oh, you don’t want to look at it, you think it’s too gross to look at” or something like that. It’s a tough situation, because at that moment he’s feeling that he’s too dirty, or that something he has is not worth looking at. … The reality is that you’re trying to set a boundary with him … .I don’t want him to lose his leg … but it’s a hard situation. I didn’t do his wound care for him, so I guess he did it somewhere privately on his own with some help from other people. It’s a tough negotiation. …

According to participants, clients were also expected to meet particular social norms, namely, to have a home with a telephone where they could be contacted, and to have the organizational ability to maintain a schedule and keep appointments. However, participants recognized that people with MHSU issues had a great deal of difficulty keeping schedules and maintaining appointments, particularly if the appointments required travel to different locations throughout the city. For marginalized clients, such expectations could intensify their feelings of alienation. Participants witnessed how clients’ frustrations erupted in moments of pain, when trying to access medications, and when appointments were missed—while other clients stared or commented.

Participants noted a variety of ways that CHCs attempt to accommodate clients who had poor experiences and found health processes alienating, especially clients who had insecure housing, were encountering law enforcement, travelling from agency to agency to obtain services, and struggling with paperwork and referrals. To address these issues, CHCs offered an assortment of services to minimize the need for travel (i.e., “one-stop shopping”), developed processes such as revised intake systems so that clients could access the resources they needed as quickly as possible, or designated people who could encourage stability in clients’ health care by maintaining a regular connection and thus engendering trust. These services aimed to decrease the frustration and anger that triggered volatility, and therefore made more stable health care possible.

Participants, however, complained about attempting to work with clients while simultaneously dealing with mainstream health care organizations. For example, a participant quoted a representative from an outside organization that excluded a client from accessing their services, saying: “This person is really behavioural; they’re not going to fit our schizophrenia study.” In another example, a participant recounted the difficulties faced by a client who was entering a long-term treatment programme, and had spent months actively preparing during day-treatment, only to be turned away at the last moment for want of space. The client “just went into a spiral … And it’s really unfortunate because we’re not reaching people in those moments.”

### Communication and stigma

People communicate stigma and discrimination in a multitude of verbal and non-verbal ways. Participants pointed out that how “we” talk about MHSU has a deep impact. A common example is that using the word “clean,” to mean “not using drugs,” implies that those who are actively using are by definition “dirty.” Indeed, providers noted the need to continually examine one’s assumptions and attitudes when thinking about language use. A peer worker remarked:

There’s enough stigma and discrimination out there. … [Clients] want information and access to services. They want help, but they don’t want to be belittled. So, if you’re going in with an attitude of ‘Poor you, I’m here to help you,’ that’s certainly not breaking down any stigma or discrimination.

A key aspect of communicating without stigma and discrimination, according to participants, is the development of excellent listening skills. This is crucial for determining what the client needs, “because they may be looking for something and asking you a completely different question” (a clinician). In addition, there is the value of hearing people’s stories, of forging social connections to understand others’ experiences and perspectives. However, being able to listen effectively requires time, which can be in dire short supply for health providers. This too can lead to frustration on the part of providers, which can exacerbate the likelihood of communicating stigma.

When health providers are communicating with clients, the potential for stigmatizing experiences is exacerbated if providers are frustrated and burned out from continually facing challenging encounters. Participants reported that their CHC’s crisis response was working very well; nonetheless, violent or aggressive interactions leave the staff on edge, and can lead to further stigma and discrimination. As a senior staff member explained:

We have had people working at the front desk who have had very bad experiences and were traumatized. … They started working here and after six months were saying, “I can’t do this anymore.

Another senior staff member remarked:

I find that a lot of the providers do tend to get burned out at certain stages, because they’re dealing with really heavy stuff that a provider in [an upper-middle class neighbourhood in Toronto] may not be dealing with. But they chose to do this type of work, and some of them go on, and continue, and love it, and some just say, ‘I think I need to do something else with my life,’ so they move to a different area.

Participants reiterated that interactions can be especially challenging if there is insufficient time, staff, support, or training. Indeed, all three CHCs struggled to meet client demand. One participant voiced concern that pressure from the Ministry of Health to increase patient loads will put further demands on clinicians who are already stressed, leading them to seek employment elsewhere, thus exacerbating staffing needs. Another explained that the Ministry’s reporting metrics do not capture the complexity of client populations in the CHCs:

… unfortunately mental health is not one of our indicators that we have to report back on. All we can say is, yes our numbers are low compared to this and that, because we deal with a very difficult-to-manage population … That’s what we have been doing, but I don’t know how much longer we can continue to sustain it. It’s an expensive venture for the government.

A related issue is having an adequately diverse staff in terms of, for example, gender or cultural background. In the midst of staffing issues, a significant challenge for providers is feeling adequately trained and supported.

### Responding pragmatically

Given the array of issues that participants regularly faced as health care providers, they described their experiences of responding as being “in the moment”; in other words, providers often found themselves focusing on the issues that caused the most immediate distress or turmoil in clients’ lives.

Participants commented that, when clients were extremely marginalized or difficult to work with, the health care approach became more about managing immediate discomfort, distress, and pain, rather than addressing root causes of illness. One senior staff member explained:

I think it takes a certain type of person who wants to do this kind of work. To be honest, you could go work in a variety of places and I think that people work in this kind of environment because they’re really committed to working with folks with multiple kinds of concerns. I think people have a very strong sense of … access and human rights … That being said I think … you have to take a bit of a different approach when you are doing this work, and I think for people that have been around for a long time, they really think of it as … more palliative kind of work … .So they have to derive some kind of satisfaction in knowing that what they’ve done is going to have an impact even though … we’re not going to see some huge dramatic kind of change in terms of somebody’s quality of life. In some instances it’s really small, incremental.

Systematic efforts to mitigate stigma were therefore inhibited by myriad proximate factors that demanded urgent response. If the provider expects different or faster outcomes, this can lead to a deep level of frustration. Participants claimed that training is key. As one clinician pointed out: “For [our health team] it’s a challenge. For them to see a client, referred by the homeless team, come to the medical appointment intoxicated. The [clinicians] say, ‘How can I deal with it? I need help.’” With respect to the availability of services, participants also commented with some frustration upon a “lack of appropriate and timely services” across Toronto, and more broadly Canada, for people with severe disorders. One described the issue in terms of:

… a [stereotypical] guy walking around in winter with no shoes … who gets taken away by the cops and goes to CAMH for 72 hours. The doctor says he’s fine, and a week later he does the same thing, and so on and so forth … until the cycle ends, generally very badly for that person.

As such, health providers at CHCs attempted to fill the gap in services for people with the most severe MHSU problems, but lacked the resources.

### Working towards practical solutions

Overall, participants were most enthusiastic about receiving further training to help clients with MHSU issues. Participants stressed the importance of integrating any training into daily health care practice, and were not interested in “one-off” training without follow-up. Although participants suggested that training should be available for all CHC personnel—from board members and managers to front-line staff and clinicians—some recommended that it be tailored to meet the specific roles they played in the organization. Some participants expressed concern about how to handle the breadth of health conditions and forms of distress confronting clients who were accessing services at the CHCs.

Another idea was to have consistent CHC-wide meetings, every month or so, to create a space where staff at all levels could get to know one another for a more communicative and inclusive organization. Such meetings were envisioned as including all staff, so that they could share their values, expertise, and ideas, acknowledging that everyone in the CHC had been hired because they were valuable to the work and the organization in some recognized way.

Participants were also enthusiastic about developing community-wide, anti-stigma education campaigns in order to ameliorate client-on-client stigma and discrimination in the CHCs, and to mitigate negative interactions within the surrounding communities. Participants noted that the accessibility of CHC programmes depends not just on how the programmes are delivered within the CHCs, but also depends on the potentially negative encounters faced in neighbourhoods *on the way* to the CHCs—whether that included being harassed because of one’s gender, being intercepted by police, or some other confrontation. Participants pointed out that in order to be effective, such a campaign would need to work with communities on multiple levels, such as local business improvement areas, social service agencies, as well as people who live in the adjacent neighbourhoods.

However, there was some discussion about how to run an anti-stigma media campaign. One participant suggested launching an advertising campaign that was “light and fluffy,” similar to the vaccination advertisements for Hepatitis A and B, in the form of TV commercials, posters on subways, or through other media. It was pointed out that such a campaign could engage an array of agencies across the city, and potentially create additional intersectoral collaborations. Regardless of the specifics of the campaign, participants commented that words such as “stigma” and “mental health” might dissuade people in the community from participating, and care should be taken with respect to the language used.

Participants were enthusiastic about implementing anti-stigma/pro-recovery approaches. Indeed, participants tended to subscribe to the notion of recovery with respect to MHSU issues. However, recovery meant different things to different people (cf. Poole, 2011). Among peer workers in this study, recovery was very much about harm reduction, for example, being respected, having access to supports, and having hope for the future despite substance use. Thus, in this context recovery did not refer to becoming “normal” or maintaining abstinence, but rather to a continuum of experiences whereby people learn to find meaning in life despite the devastating setbacks associated with MHSU problems (Anthony, 1993). Other health providers wanted recovery-based approaches to be more strongly integrated into ongoing CHC practices, and contextualized recovery in relation to the limitations of the services they could provide. As one clinician remarked:

We don’t do [enough to] support mental health throughout the spectrum. We do a lot of putting out fires … .We see people through the stages of recovery and see them with good outcomes. [But too often] we sort of get to “good enough” which [means] you’re housed, you’re on medication and you’re sort of stable. Most of the people who are like that that I have as clients aren’t happy, they’re unfulfilled. They’re not working, they’re not looking forward to their lives … We don’t help them move past that.

Another clinician commented:

The stigma is, if I had a patient who had broken their leg and I’d want the cast off eventually, I’d want to stop using the crutches and I’d want them to be doing their marathons again. But with mental health, we have this stigma that the end point is less than. Like, 60% is okay. We don’t have that philosophy here that anyone who has mental health [problems] should be happy and engaged in their lives and fulfilled, and maybe having kids and families if that’s what they want … we just don’t have any planning for them ourselves.

It is important to underscore, then, that recovery is perceived and experienced along a continuum of meanings according to the client’s history and context.

It is important to emphasize that any efforts to build an anti-stigma intervention would not occur in a vacuum; CHC staff have already done a great deal of work to decrease the stigma and discrimination associated with MHSU. What is working well at the different CHCs? Participants reported that the development of central intake systems at two of the CHCs has been key for minimizing stigmatizing experiences while getting help to clients quickly. Team approaches to care, and using case conferencing to coordinate care and work through any disagreements between providers, have been instrumental as well. They also indicated that work had been done with marked success to educate CHC staff about the value of harm reduction approaches, that harm reduction clients are as important as others, and that harm reduction workers do not enable illegal substance use. As indicated above, CHC staff for whom MHSU issues were not their “core work” sometimes held such stigmatizing beliefs, which then needed to be addressed in order to avoid alienating marginalized clients.

Programmes for specific client groups were described by peer workers as reflective or representative of communities, and were said to be working well because they were culturally or socially relevant (e.g., programmes for First Nations and other ethnic groups, youth and seniors’ programmes, community kitchens, women’s programmes including drop-in services and services for sex-workers, celebrations such as for Earth Day, and the like). Participants described the intensive work involved in community outreach; this helps to build relationships and engender trust with community members, which is often a necessary first step to get clients into counselling or primary care. Other efforts such as educating communities about harm reduction, community sweeps and clean-up, offering different programmes such as legal assistance and housing, and hiring peer workers, have been effective in decreasing the stigma of MHSU.

## Discussion

Our findings speak to the complexities of and opportunities for improving the health care experiences of marginalized people at CHCs in urban Toronto. More specifically, we sought to explore the issues related to stigma and discrimination in these locations, with a view to creating effective anti-stigma/anti-discrimination capacity-building programmes. While positive outcomes have been documented for anti-stigma interventions that target specific groups and for mass media campaigns, challenges remain for assessing the effects of anti-stigma efforts over the long term (Gronholm et al., 2017). Increasing efforts are underway to develop an implementation science for assessing the suitability, feasibility, as well as the short- and long-term effectiveness of anti-stigma interventions (Kemp et al., 2019). PHC settings offer unique opportunities to address stigma and discrimination relating to MHSU problems (Sapag et al., 2012). However, few studies have focused on designing and implementing interventions aimed at reducing stigma and discrimination among health providers within PHC contexts (Shim & Rust, 2013).

In this study, participants emphasized the importance of contextual complexities and the interplay of multiple stigmas; MHSU problems occur in a broader context of poverty, insecure housing and food insecurity, gender and age bias, racism and ethnocentrism, language barriers, poor access to training and jobs, and marginalization based on employment (e.g., sex work). Participants also discussed MHSU problems in the context of trauma due to family abuse, governmental intervention (e.g., residential schools), and violence being encountered currently in the streets.

With respect to client encounters with health providers, participants reported that stigma and discrimination can be exacerbated by cultural misunderstandings. Clients may also encounter barriers or experiences of alienation because of the way that mainstream health systems are structured; for example, people with MHSU problems can have difficulties maintaining appointments, navigating different health services across the city, or exhibiting certain behaviours in certain spaces. Stigma and discrimination can occur because of the language used to discuss MHSU, or because a health provider is burned out from dealing with ongoing crises, or with clients who are aggressive or have high levels of need.

Participants identified a number of opportunities for action. Health providers were most enthusiastic about receiving additional and ongoing training; however, they noted that they were not interested in “one-off” sessions without follow-up but in training that could be integrated into practice on an ongoing basis. Various kinds of training were suggested and, in the focus groups, debated. Recommendations included exploring MHSU issues over the life course. Another recommendation was to hold monthly or bi-monthly CHC-wide meetings, in order to help build a positive organizational culture by offering a space for inclusive communication amongst staff at all levels of the CHC. Participants also advocated conducting anti-stigma/anti-discrimination campaigns in local communities, by building on existing linkages that the CHCs have forged within their neighbourhoods. Such campaigns would need to target people at multiple levels, such as business owners, social service agencies, as well as those living in neighbourhoods surrounding the CHCs.

### Study limitations

The authors are aware that it is important to explore stigma and discrimination relating to MHSU problems with CHC clients who are not peer workers as well as with other community members. Addressing this limitation, the research team undertook a follow-up study with these groups, the findings of which can be found elsewhere (Murney et al., 2014).

A potential limitation in the current study exists in that focus group participants and peer workers were suggested by the CHC liaison representatives, in a case of purposive sampling. In quantitative work, this would introduce a problem of selection bias. However, as is common with qualitative inquiries, this sampling strategy is an asset, for it enabled the authors to seek information-rich cases in order to maximize description and depth (cf. Patton, 2002, pp. 230–246). The possibility also exists for social desirability bias (Link et al., 2004; Phillips & Clancy, 1972) in that participants may have provided answers on the basis of what they assumed the researchers would regard as favourable. Ethnographic work would have ameliorated this issue, but there was a lack of funding and time for this intensive data collection method. The first author, who conducted the interviews and focus groups, was attentive to this limitation, and thus took extra time to build rapport with participants.

## Conclusion

Stigma and discrimination have wide-ranging effects both within and outside of health care settings. This is one of the first studies of MHSU-related stigma and discrimination in PHC settings in Canada. Taking a phenomenological approach allowed us to emphasize or foreground the lived experience and interpretations of health providers who, on a daily basis, witness the unfolding of myriad forms of stigma and discrimination, while seeking to address stigma and discrimination. Our findings highlight the complex health needs of CHC clients, the challenges that health providers face within CHCs, as well as possibilities for enhancing both health care access and experiences for some of Ontario’s most marginalized people. Moreover, this research focused on CHCs in Toronto, health centres in an extremely diverse urban setting, which often serve marginalized clients who experience multiple, intersecting stigmas. As such, this research may be useful for informing health planners and policymakers about the complexities surrounding MHSU-related stigma and discrimination in PHC settings.

Finally, it is crucial to respond to these challenges by creating effective anti-stigma/anti-discrimination capacity-building programmes. The authors expect that the results will be useful for designing future training and health initiatives that integrate knowledge about stigma, discrimination, and MHSU issues, with particular attention given to immigrant and other marginalized populations, in PHC settings in Canada. Further research is needed to inform the design of capacity-building initiatives, as well as research to evaluate the long-term effectiveness of such programmes for improving health outcomes and access to health care.
